# Exposure to and Engagement With Digital Psychoeducational Content and Community Related to Maternal Mental Health by Perinatal Persons and Mothers: Protocol for a Web-Based Survey With Optional Follow-Up

**DOI:** 10.2196/64075

**Published:** 2025-04-30

**Authors:** Molly E Waring, Katherine E McManus-Shipp, Christiana M Field, Sandesh Bhusal, Asley Perez, Olivia Shapiro, Sophia A Gaspard, Cindy-Lee Dennis

**Affiliations:** 1 Department of Allied Health Sciences University of Connecticut Storrs, CT United States; 2 Department of Psychological Sciences University of Connecticut Storrs, CT United States; 3 Lawrence S. Bloomberg Faculty of Nursing University of Toronto Toronto, ON Canada; 4 Lunenfeld-Tannenbaum Research Institute Mount Sinai Hospital Toronto, ON Canada; 5 Department of Psychiatry University of Toronto Toronto, ON Canada

**Keywords:** social media, podcasts, blogs, perinatal mental health, maternal mental health, digital health, engagement

## Abstract

**Background:**

Leveraging digital platforms may be an effective strategy for connecting perinatal persons and mothers with evidence-based information and support related to maternal mental health and peers. Momwell is a mom-centered model of care that provides psychoeducational content through several digital platforms, including social media, a podcast, and a blog. The aims of this project were to describe how perinatal persons and mothers engage with Momwell’s psychoeducational content and community; describe the perceived benefits of exposure to and engagement with content and community; examine associations between engagement with digital psychoeducational content and maternal mental health, parenting attitudes, and interparental relationships; and examine changes in mental health and parenting attitudes and concurrent engagement with Momwell’s digital psychoeducational content and community over 2 to 3 months.

**Objective:**

This paper aims to describe the design of a study of perinatal persons and mothers who are exposed to or engage with Momwell’s psychoeducational content and community and describe sample characteristics.

**Methods:**

Adults who engaged with Momwell on any of their digital platforms were recruited to complete a web-based survey in July 2023 to September 2023. Participants completed either a longer or shorter survey. Participants who provided permission to be recontacted were invited to complete a second survey 2 to 3 months later. The surveys included validated psychological measures, study-specific quantitative questions, and open-ended questions that assessed participant demographics, exposure to and engagement with Momwell’s psychoeducational content and community, maternal mental health, parenting relationships, parenting self-efficacy, and additional psychosocial and health measures. We outline planned analyses to achieve the aims of the project.

**Results:**

Data collection occurred from July 2023 to September 2023 (N=584). A subset of participants completed the optional second survey in October 2023 to December 2023 (N=246). Participants were >99% mothers (582/584, 99.7%); 45.5% (266/584) perinatal (59/584, 10.1% pregnant; 210/584, 36% post partum); and, on average, aged 32.4 (SD 3.9) years. In total, 59.1% (345/584) were from the United States, 35.6% (208/584) were from Canada, and 5.3% (31/584) were from other countries. The vast majority (552/584, 94.5%) followed Momwell on Instagram, 44.2% (258/584) listened to the Momwell podcast, and 41.1% (240/584) received their newsletter. Most participants had been exposed to Momwell’s psychoeducational content for at least 6 months across the different platforms (range 16/36, 44% on TikTok to 480/552, 87% on Instagram).

**Conclusions:**

Data from this study will provide insights into how pregnant persons and mothers use digital psychoeducational content and peer communities to support their mental health throughout the perinatal period and into the early years of motherhood. Leveraging digital platforms to disseminate evidence-based digital psychoeducational content related to maternal mental health and connect peers has the potential to change how we care for perinatal persons and mothers.

**International Registered Report Identifier (IRRID):**

DERR1-10.2196/64075

## Introduction

### Background

Depression and anxiety are common during pregnancy and the postpartum period, yet many struggling with their mental health do not seek mental health care [1,2]. Mothers often feel pressure to achieve socially constructed ideals of motherhood and struggle to balance life roles and responsibilities, which together can negatively impact their mental health [2,3]. Prevention, screening, and treatment of perinatal mental health problems are critical given the long-lasting impact that perinatal mental health can have on mothers, children, and families. Several meta-analyses and systematic reviews have shown that psychosocial, psychological, and psychoeducational interventions can improve mood and reduce the risk of depression among perinatal persons [4-6] and parents of young children [7]. A recent meta-analysis found that peer support interventions are efficacious for preventing or managing perinatal and postpartum depression [8]. Specifically, information and support from peers via phone can prevent postpartum depression in high-risk women [9], and online asynchronous support groups can also reduce depressive symptoms during the perinatal period [10].

Leveraging digital platforms such as social media and podcasts may be an effective strategy for overcoming barriers to seeking mental health care and connecting perinatal persons and mothers with evidence-based information and peer support related to maternal mental health [11]. Most women of childbearing age and mothers use social media, including those in Canada [12] and the United States [13-15], and many turn to their online networks for support and information about diverse health and parenting topics [16,17]. In addition, 67% of adults in the United States aged 18 to 39 years and 58% of those aged 30 to 49 years listen to podcasts [18]. Connecting with other perinatal persons or mothers online can decrease feelings of isolation, provide a safe space to discuss mental health without feeling judged or stigmatized, and increase parenting confidence [19,20]. Given the ever-expanding opportunities for psychoeducation and peer support available via social media and other digital platforms such as podcasts, content disseminated via these digital platforms has the potential to reach millions for widespread impact on perinatal and maternal mental health. However, little is known about how perinatal persons and mothers consume and engage with psychoeducational content disseminated through digital platforms and how this psychoeducational content and peer interactions impact perinatal and maternal mental health. There is an ongoing need to understand how people access and use digital mental health resources focused on perinatal and parenting populations. We conducted a web-based survey of adults exposed to or who engage with Momwell via social media or other digital platforms to address this gap.

### Objectives

The overarching aims of this project are to (1) describe how perinatal persons and mothers engage with Momwell’s psychoeducational content and community; (2) describe the perceived benefits of exposure to and engagement with Momwell’s psychoeducational content and community; (3) examine associations between engagement with digital psychoeducational content and maternal mental health, parenting attitudes, and interparental relationships; and (4) examine changes in mental health and parenting attitudes and concurrent engagement with Momwell’s digital psychoeducational content and community over 2 to 3 months. In this paper, we describe the design of the study and the characteristics of the sample. As most women of childbearing age and mothers in the United States and Canada use social media [12-15] and listen to podcasts [18], there is great potential for wide dissemination of relevant and impactful evidenced-based psychoeducational content that can support maternal mental health.

## Methods

### Overview

We recruited a convenience sample of adults exposed to or who engage with Momwell via social media or other digital platforms to complete a web-based survey. We invited participants who provided permission for us to recontact them to complete a second survey 2 to 3 months later.

### Ethical Considerations

The University of Connecticut Institutional Review Board approved this study (protocol X23-0344). Individuals were recruited to participate in 1 of 2 cohorts (cohort 1 and cohort 2); participants in cohort 2 completed shorter surveys at both time points. Before completing the survey, participants were shown a cohort-specific information sheet and provided with an opportunity to download a PDF of the information sheet. Participants consented to the research study electronically before completing their survey. We stored study data on password-protected research drives. Only appropriate research staff had access to study data on Qualtrics (Qualtrics LLC), REDCap (Research Electronic Data Capture; Vanderbilt University), or research drives. Cohort 1 participants who completed at least 80% of the survey received a gift card (US $15 or CAD $20 [US $14.47-$15.35 over course of study]). Cohort 2 participants who completed at least 80% of the survey were entered into a gift card lottery. One in every 30 participants was randomly selected to receive a gift card (US $60 or CAD $80 [US $57.88-$61.40 over course of study]). Cohort 1 participants who completed at least 80% of the optional second survey received a gift card (US $15 or CAD $20 [US $14.47-$15.35 over course of study]), and cohort 2 participants who completed at least 80% of their survey were entered into a gift card lottery in which 1 in 30 participants was randomly selected to receive a gift card (US $60 or CAD $80 [US $57.88-$61.40 over course of study]).

### Recruitment and Eligibility Screening

From July 2023 to September 2023, we recruited adults who followed Momwell on any of their social media platforms, listened to their podcast, read their blog, or received their email newsletter. Founded and led by Erica Djossa, a registered psychotherapist based in Ontario, Canada, Momwell offers a mom-centered model of care that seeks to educate, empower, and support mothers. Momwell offers individual telehealth psychotherapy to clients in Canada and the United States and self-paced virtual workshops and courses led by Ms Djossa and her team of licensed psychotherapists. In addition to these for-fee services, Momwell provides evidence-based psychoeducational content about motherhood, parenting, and maternal mental health to the public without cost through their social media feeds (Instagram, Facebook, and TikTok), weekly podcast, and blog with posts corresponding to the podcast episodes. A weekly email newsletter alerts readers to new podcast episodes and blog posts and provides other updates, including new workshop and course offerings. In this study, we focused on exposure to and engagement with the Momwell psychoeducational content and community via their social media feeds, podcast, blog, and email newsletter (ie, resources freely available to the public).

The Momwell team posted recruitment messages that included a link to an eligibility screener via Qualtrics. Recruitment messages were posted as public social media posts and Instagram stories. On Instagram, interested individuals could comment “STUDY” to be sent a link to the eligibility screener via direct message. An advertisement was included in a Momwell podcast in mid-July 2023. In addition, a message about the study was included in Momwell’s weekly email newsletter. These recruitment messages included a link to the eligibility screener; this eligibility screener could be considered an open survey in that any individual with the link could complete the screener. Inclusion criteria for both cohorts included age of ≥18 years, following or engagement with Momwell on at least one of their digital platforms (eg, Instagram, Facebook, Facebook group, TikTok, podcast, blog, or email newsletters), comfort participating in the study in English, and ability and willingness to provide informed consent. To participate in cohort 1, individuals additionally had to be (1) a perinatal person (eg, currently pregnant or within 12 months post partum) or mother (eg, identifying as the mother of at least one child aged <18 years who lived with them at least part time) and (2) currently living in Canada or the United States. As part of the eligibility screener, respondents indicated whether they had a preference for completing the longer or shorter survey (or had no preference); participants who expressed preference for the shorter survey were all invited to complete it.

As some recruitment messages were posted publicly, we recognized the potential for attracting the attention of bots or individuals trying to participate under false pretenses to receive the gift card incentive [21-24]. As recommended [23,24], we used a multifaceted strategy to ensure that enrolled participants were truly eligible, including Qualtrics bot and fraud detection features, checking for duplicate respondents (eg, email and IP address), and checking the consistency of the information (eg, specific recruitment link clicked on vs where the respondent reported they had heard about the study). Individuals whose responses to the eligibility screener suggested that they were a bot or fraudulent were not invited to complete the survey.

### Data Collection

Eligible individuals were emailed personal invitations to complete a web-based survey via Qualtrics. Individuals who did not respond to the invitation were sent a reminder 1 day later. Nonresponders were sent 2 additional invitations a few weeks later. Participants who partially completed the survey were sent a reminder email with a new survey link. The survey included both quantitative and open-ended questions. The survey for cohort 1 participants was designed to take 30 to 45 minutes to complete (median 52, IQR 36-95 minutes among those who completed at least 80% of the survey). The survey for cohort 2 participants was a shortened version of the cohort 1 survey comprising a subset of questions and scales. It was designed to take 15 to 20 minutes to complete (median 29, IQR 21-45 minutes among those who completed at least 80% of the survey).

We reviewed participants’ responses and survey metadata to detect likely fraudulent respondents who were not detected at eligibility screening (eg, discrepancies between metadata and survey responses and discrepancies between data provided in the eligibility screener and survey). As recommended [23,24], we conducted additional data quality checks at this phase of data collection; we reviewed the surveys for patterns of responses indicating inattentiveness or fraudulent responses (eg, completing the survey in less than half the median time for that cohort; straight-lined psychological measures with reversed items; failure of an attention-check item; or missing, extremely brief, or nonsensical responses to open-ended questions). We contacted participants with multiple flags via phone or email to clarify their responses or ask them to provide responses to the open-ended survey questions. Individuals whom we could not reach or who provided conflicting responses were excluded. Our final analytic sample includes participants who completed at least 80% of their survey who were not excluded during the data quality review.

The final question in the survey asked participants for permission to contact them about future research studies, specifically, a second survey approximately 2 months later. Participants who responded affirmatively were invited to complete an optional second survey 2 to 3 months later (October 2023-December 2023). Study procedures (eg, survey invitations, reminders, and consent process) were the same as procedures for the main survey. Survey response review was also similar to that of the main survey; we additionally compared consistency of responses between the 2 surveys (eg, pregnancy or postpartum status of participants who were pregnant when they completed the main survey). Similar to the main survey, the second surveys were designed to take 30 to 45 minutes (cohort 1; median duration 34, IQR 24-62 minutes among those who completed at least 80% of the survey) or 15 to 20 minutes (cohort 2; median duration 12, IQR 9-22 minutes among those who completed at least 80% of the survey).

To ensure that participants had opportunities to be exposed to and engage with content related to perinatal mental health, the Momwell content development team created and published 3 psychoeducational posts specifically related to perinatal mental health per week for 16 weeks beginning at the start of recruitment for the main survey.

### Measures

#### Overview

The surveys included a rich set of validated psychological measures, study-specific quantitative questions, and open-ended questions that assessed exposure to and engagement with Momwell’s psychoeducational content and community, maternal mental health, parenting relationships, parenting self-efficacy, and other participant characteristics and behaviors. The measures are described in the following sections and in Table 1. The study team tested the surveys, including survey logic before recruitment and data collection. The number of questions asked of each participant varied depending on their responses to previous questions (eg, only participants who followed Momwell on Instagram were asked questions about their exposure to and engagement with Momwell content on Instagram). Participants were not able to go back to previous survey pages once submitted (ie, the back button on the survey was disabled).

**Table 1:** Measures assessed by cohort and time point.

Measure			Main survey					Optional second survey		
			Cohort 1		Cohort 2		Cohort 1			Cohort 2
**Exposure to and engagement with the Momwell psychoeducational content and community**										
	Exposure to and engagement with the Momwell content and community	✓		✓		✓			✓	
	Perceived impact of Momwell content	✓		✓		✓			✓	
	Suggestions to increase engagement and desired topics for future posts or podcast episodes	✓		✓						
**Mental health**										
	PHQ-8^a^ [25,26]	✓		✓		✓			✓	
	GAD-7^b^ [27]	✓		✓		✓			✓	
	PSS-10^c^ [28]	✓		✓		✓			✓	
**Parenting support and self-efficacy**										
	PPSS^d^ [29]	✓				✓				
	BCRS^e^ [30]	✓				✓				
	PSOC^f^ [31]	✓				✓				
**Additional psychological measures**										
	TIPI^g^ [32]	✓		✓						
	GHSQ^h^ [33,34]	✓				✓				
	MHLS^i^ [35]	✓				✓				
	Brief COPE^j^ [36]	✓				✓				
	INCOM^k^ [37-39]	✓				✓				
	SCS-SF^l^ [40,41]	✓				✓				
	ULS-8^m^ [42,43]	✓				✓				
**Additional participant characteristics**										
	General social media use and digital health literacy	✓		✓						
	Demographics and household composition	✓		✓		✓			✓	
	Health and health behaviors	✓		✓		✓			✓	
	Health care use	✓		✓		✓			✓	

^a^PHQ-8: 8-item Patient Health Questionnaire.

^b^GAD-7: 7-item Generalized Anxiety Disorder scale.

^c^PSS-10: 10-item Perceived Stress Scale.

^d^PPSS: Postpartum Partner Support Scale.

^e^BCRS: Brief Coparenting Relationship Scale.

^f^PSOC: Parenting Sense of Competence Scale.

^g^TIPI: Ten-Item Personality Inventory.

^h^GHSQ: General Help-Seeking Questionnaire.

^i^MHLS: Mental Health Literacy Scale.

^j^COPE: Coping Orientation to Problems Experienced.

^k^INCOM: Iowa-Netherlands Comparison Orientation Measure.

^l^SCS-SF: Self-Compassion Scale–Short Form.

^m^ULS-8: University of California, Los Angeles, Loneliness Scale.

#### Exposure to and Engagement With Momwell’s Psychoeducational Content and Community

As part of the eligibility screener, main survey, and second survey, participants were asked a variety of questions about their exposure to and engagement with Momwell’s psychoeducational content and community to capture different dimensions of exposure and engagement, including duration, frequency, and depth of active engagement (Table 1).

In the eligibility screener, participants were asked whether they listened to the Momwell podcast; followed Momwell on Instagram, Facebook, or TikTok; were members of the Momwell Community Facebook group; followed the Momwell blog; or received the Momwell email newsletters. From this, we categorized participants as social media followers (ie, followed Momwell on Instagram, Facebook, or TikTok or were members of the Momwell Facebook group) or not. We also categorized whether participants listened to the Momwell podcast or read the Momwell blog as both these platforms provide a deeper dive into psychoeducational topics and blog posts are published that correspond to each podcast episode.

Those reporting exposure to content on each platform were asked how recently they had started consuming content from that platform (“within the past 7 days,” “at least 7 days ago but less than 3 months ago,” “at least 3 months ago but less than 6 months ago,” “at least 6 months ago but less than 12 months ago,” and “at least 12 months ago”). As Momwell rebranded themselves as “Momwell” from “Happy as a Mother” in January 2023, we prompted participants to include involvement with “Happy as a Mother.” To capture duration of exposure to Momwell content, we calculated the longest duration of exposure on any platform (ie, how long ago they had joined via their first digital platform). We also calculated how recently they had joined a new platform.

For each platform, participants reported how often they engaged in specific ways applicable to that platform within the previous 4 weeks (or since they had started following Momwell on that platform if more recently than 4 weeks before) on a 4-item Likert scale (“every day,” “3+ times per week but not every day,” “1-2 times,” and “not at all”). Activities included passive exposure to content (eg, reading posts, watching videos, listening to podcast, and reading the blog) and more active and visible forms of engagement (eg, liking, reacting, and replying to posts).

Participants who completed the second survey who did not report exposure to Momwell content on a particular platform at eligibility screening were asked about exposure to Momwell content on that platform during the second survey. Existing and new users of each platform were then asked the same questions about their exposure to and engagement with the Momwell content and community since they completed the main survey (eg, “since you completed the first survey in August 2023”).

In the main survey, participants were asked several questions related to their perceived impact of Momwell content on their lives. First, they were asked the following: “What Momwell social media post, blog post, or podcast has had the biggest impact on your life? How did it impact you?” In the second survey, they were asked similar open-ended questions about the perceived impact of Momwell content but specific to each platform. In both surveys, participants were also asked whether they had taken several actions as a result of their exposure to Momwell content (eg, “talked with a doctor or health care professional about something you heard in the Momwell podcast or saw on a social media post”). In the main survey, participants reported the extent to which they agreed with several statements about changes since joining the Momwell community (eg, “I feel more aware of the signs and symptoms of mental health conditions or I feel more confident in my approach to parenting”). In the main survey, participants were also asked what would help them engage more with the Momwell community and what topics or content they would like to see more of from Momwell.

While our primary interest was in exposure to and engagement with the free psychoeducational content and peer community provided by Momwell through their social media feeds, podcast, and blog, we also asked participants about paid services available from Momwell. Specifically, we asked participants who had joined the Momwell community >7 days before whether they had ever purchased a guided journal or other self-paced tool from Momwell, enrolled in a Momwell workshop or course, or enrolled in therapy (in person or remote) with a Momwell therapist. From the responses, we calculated the proportion who had purchased any paid services.

#### Mental Health

Our main measures of maternal mental health are depression, anxiety, and perceived stress. Participants in both cohorts were asked to complete these measures in the main and second surveys (Table 1). Depressive symptoms were assessed using the 8-item Patient Health Questionnaire [25,26]. This scale asks participants to indicate on a 4-point scale how often they have been bothered by various depressive symptoms in the past 2 weeks. Response options include “Not at all,” “Several days,” “More than half the days,” and “Nearly every day.” Responses are summed to obtain a total score ranging from 0 to 24, with a greater score indicating greater reported depressive symptoms. Internal consistency was acceptable, with Cronbach α=0.85 in the main survey and Cronbach α=0.86 in the second survey compared to the original measure (Cronbach α=0.86-0.89 across samples) [25].

Symptoms of anxiety were assessed using the 7-item Generalized Anxiety Disorder scale [27]. Participants are asked to report how often they have been bothered by symptoms of anxiety over the past 2 weeks. Response options include “Not at all,” “Several days,” “More than half the days,” and “Nearly every day.” Responses are summed to obtain a total score ranging from 0 to 21, with a greater score indicating higher symptoms of anxiety. Internal consistency was acceptable, with Cronbach α=0.91 in the main survey and Cronbach α=0.90 in the second survey compared to the original measure (Cronbach α=0.92) [27]. Participants also reported whether they had ever been diagnosed with depression or anxiety during pregnancy, the postpartum period, or a nonperinatal period of life. Participants reporting a history of depression (or anxiety) were asked whether they currently had depression (or anxiety).

Participants who scored ≥10 on the 8-item Patient Health Questionnaire or 7-item Generalized Anxiety Disorder or who endorsed feeling as if they were currently experiencing depression or anxiety were flagged and shown a message noting that they might be experiencing some feelings of depression (or anxiety depending on which was flagged); encouraging them to connect with a mental health care professional for assessment, support, and therapy if appropriate; and noting that their primary care provider, obstetrician, or gynecologist may also be a good resource for support or to help them connect with a mental health care professional. Participants were then offered the option of downloading a PDF mental health resource guide developed by the research team (options for participants from the United States, Canada, and other countries). Participants were asked whether they wanted a copy of the guide sent to them via email, and research staff emailed a copy of the appropriate mental health guide to participants who answered affirmatively.

The 10-item Perceived Stress Scale [28] was used to assess perceived stress during the past 2 weeks. Participants selected how often they experienced the feelings and thoughts presented in the items via response options ranging from “Never” to “Very often.” Responses are summed to obtain a total score with a range from 0 to 40, with a higher score indicating higher perceived stress. Internal consistency was acceptable, with Cronbach α=0.88 in the main survey and Cronbach α=0.88 in the second survey compared to the original measure (Cronbach α=0.78) [28].

#### Parenting Support and Self-Efficacy

Participants in cohort 1 completed a set of measures related to parenting support and self-efficacy (Table 1). We first asked participants with children whether they had a parenting partner (ie, a partner who assisted the participant with parenting, with a note that, for many people, their parenting partner is their spouse or the child’s other parent).

Participants who reported having a parenting partner completed a modified version of the Postpartum Partner Support Scale [29], a validated 20-item measure that assesses support provided by their husband or partner. To better represent a variety of partnerships and family structures, we modified the Postpartum Partner Support Scale to replace “him” (ie, husband) with “my partner.” We also modified questions to refer to “our children” rather than “our baby.” Responses are summed to obtain a total score with a range from 20 to 80, with higher scores indicating higher parenting support. Internal consistency was acceptable, with Cronbach α=0.95 in the main survey and Cronbach α=0.96 in the second survey compared to the original measure (Cronbach α=0.96) [29].

Participants who reported a parenting partner completed a modified version of the 14-item Brief Coparenting Relationship Scale [30] to assess interparental relationships. We modified the phrasing of the items and measure instructions to refer to “child(ren)” versus “child” to better reflect families of various sizes. Responses are averaged, with scores ranging from 0 to 6 and higher scores indicating a more positive relationship with the parenting partner. Internal consistency was acceptable, with Cronbach α=0.90 in the main survey and Cronbach α=0.91 in the second survey compared to the original measure (Cronbach α=0.81-0.89 across different samples) [30].

Perceived parenting competence was assessed using a modified version of the 16-item Parenting Sense of Competence Scale (PSOC) [31]. We modified the language of the PSOC to replace the words “mother” and “father” in items with “parent,” as well as to replace “child” with “children.” The PSOC is composed of 2 subscales, efficacy and satisfaction, which combined produce an overall score representing parenting self-esteem. The overall PSOC score is reflective of 16 items, with a range from 16 to 96 and higher scores indicating greater sense of overall parenting self-esteem. The satisfaction subscale comprises 9 items, with scores ranging from 9 to 54. Higher scores indicate higher levels of parenting satisfaction. The efficacy subscale is assessed using 7 items, with scores ranging from 7 to 42 and higher scores indicating more parenting efficacy. Internal consistency was acceptable for the overall score and both subscales, with Cronbach α=0.80 for the overall score, Cronbach α=0.77 for the satisfaction subscale, and Cronbach α=0.76 for the efficacy subscale in the main survey and Cronbach α=0.83 for the overall score, Cronbach α=0.77 for the satisfaction subscale, and Cronbach α=0.83 for the efficacy subscale in the second survey compared to the original measure (Cronbach α=0.79 for the overall score, Cronbach α=0.76 for the satisfaction subscale, and Cronbach α=0.76 for the efficacy subscale) [31].

#### Additional Psychological Measures

We included several additional psychological measures to provide data for secondary analyses (Table 1). As part of the main survey, participants in both cohorts completed the Ten-Item Personality Inventory [32], which assesses the personality traits extraversion, agreeableness, conscientiousness, emotional stability, and openness to experience. Participants in cohort 1 completed several additional measures as part of the main and second surveys (Table 1). Participants completed a modified General Help-Seeking Questionnaire [33,34], which asked individuals to rate how likely they would be to seek help from various sources (eg, partner or spouse and mental health professional) if they were experiencing sadness, low mood, irritability, and other depressive symptoms. Knowledge of mental health conditions and mental health literacy were assessed using a subset of questions from the Mental Health Literacy Scale [35]. Given the interests of this study (perinatal mental health vs general mental health or illness), we included items assessing knowledge of generalized anxiety disorder and major depressive disorder, the connection between sleep and mood, information seeking related to perinatal depression, and general attitudes toward people with perinatal depression (stigma).

Participants completed the Iowa-Netherlands Comparison Orientation Measure [37-39], which is designed to assess frequency of social comparisons. We modified the measure stem to ask about comparisons with “other mothers” rather than “other people” to assess the tendency to make social comparisons to other mothers. Participants completed the short form version of the Self-Compassion Scale [40,41], a validated questionnaire measuring self-compassion via 6 subscales. Feelings of loneliness were ascertained using the short-form Revised University of California at Los Angeles Loneliness Scale [42,43]. Finally, participants completed the Brief Coping Orientation to Problems Experienced [36], which assesses 14 coping strategies: self-distraction, active coping, denial, substance use, use of emotional support, use of instrumental support, behavioral disengagement, venting, positive reframing, planning, humor, acceptance, religion, and self-blame.

#### General Social Media Use and Digital Health Literacy

Participants reported their use of social media generally, including whether they had accounts on different platforms (ie, Instagram, Facebook, or TikTok); how often they engaged with various platforms in different ways (eg, posting a video); and whether they followed social media accounts related to motherhood or parenting, mental health, or another health topic. Participants were also asked whether they subscribed to or regularly listened to podcasts (other than Momwell). We modified 2 items from the eHealth Literacy Scale [44] to assess perceived skill and confidence using social media to access health information: “I have the skills I need to evaluate health information I see on social media” and “I feel confident in using health information from social media to make health decisions for me or my family.” Response options ranged from “Strongly Disagree (1)” to “Strongly Agree (5).” As a brief screener of health literacy, participants were asked how confident they were filling out medical forms by themselves [45].

#### Demographic Characteristics, Health Behaviors, and Health Care Use

Participants reported demographic characteristics, including country and state or province of residence, urbanicity of their city or town of residence, gender, sex assigned at birth, and sexual orientation [46]. Race and ethnicity were assessed via an open-ended question (ie, “How would you describe your racial and ethnic background?”). Participants living in the United States and Canada were also asked to indicate their racial and ethnic background by choosing all that applied from a standardized list corresponding to each country. Participants from the United States were also asked whether they considered themselves Hispanic or Latino (yes or no). Participants also reported whether they self-identified as someone who was a visible minority [47]. Participants also reported educational level, employment status, family structure, the number of children living in the home at least part time, and the ages of those children. In the second survey, participants were asked about current pregnancy (including gestational age); whether they had given birth to a baby in the past 12 months; and, if yes, how many months before they had given birth. Financial strain was assessed by asking participants to identify how difficult it had been for them, in the past 30 days, to pay for usual household expenses (eg, food, rent, car payments, or medical expenses) [48]. Participants reported their current employment status by selecting one of the following options: “working full-time,” “working part-time,” “working but currently on parental leave,” “stay-at-home parent or homemaker,” “unemployed,” “student,” and “other.” Given the small number of participants who endorsed each of these categories, we collapsed “unemployed,” “student,” and “other” into 1 category.

Participants completed the Hunger Vital Sign [49], a validated 2-item food insecurity screening tool based on the US Household Food Security Survey Module. Participants reported whether, in the past 12 months, they had ever worried about food running out due to financial hardship and whether there was ever not enough money to get more food when food did run out. Participants who answered either of the 2 items with “often true” or “sometimes true” (vs “never”) were considered to be at risk of experiencing food insecurity [49]. Participants who completed the second survey reported employment status, food security, financial strain, and changes to the household structure occurring since completing the main survey. To put any changes in mental health in context, as part of the second survey, we asked participants to report whether they had experienced various life events since the first survey (eg, spouse or partner dying, getting divorced or separated, becoming pregnant, giving birth, or adopting a child). Life events were adapted from the Holmes-Rahe Life Stress Inventory [50]; we added “gave birth or adopted a child.” Participants who reported one or more potentially stressful life events were asked to rate how stressful that time was for them on a Likert scale from “not at all stressful” to “extremely stressful.”

Participants were asked questions about their health, health behaviors, and health care use. Participants self-reported their height and weight and whether they had any physical or mental health conditions requiring frequent medical visits (and, if so, what conditions). Participants answered questions regarding diet, alcohol consumption, tobacco use, physical activity, and sleep. To assess diet quality, participants completed the 8-item Starting The Conversation dietary screener [51]. Participants reported how many days per week they consumed at least one alcoholic beverage, the number of alcoholic drinks per day they consumed, and the frequency of binge drinking. Participants also reported ever use and current use of cigarettes and e-cigarettes. Participants reported frequency and duration of moderate or greater-intensity physical activity in the past 4 weeks. To assess quality of sleep in the previous 4 weeks, participants completed a subset of questions from the Pittsburgh Sleep Quality Index [52] related to sleep duration and self-rated sleep quality. Participants first reported via free response how many hours they slept at night. Participants were then asked to rate their quality of sleep, with possible answer options of “very bad,” “fairly bad,” “fairly good,” and “very good.” Mothers were also asked how often in the past 4 weeks a child had disrupted their sleep. Participants reported any physical or mental health care services accessed via in-person visits, telehealth visits, phone call, email, or online patient portal in the past 6 months. In the second survey, participants were asked about new medical diagnoses, health care use, and sleep.

### Statistical Analysis

The eligibility screener and surveys were administered in Qualtrics. We used REDCap for participant tracking [53]. Analyses were conducted in SAS (version 9.4; SAS Institute). We described recruitment and enrollment. As recommended when reporting the results of a web-based survey [54], we calculated the participation rate as the number consented divided by the number invited to participate and the completion rate as the number who completed the survey (ie, up to the last page) divided by the number consented for both the main and optional second surveys. We described the characteristics of the participants in our final sample overall and by study cohort. We compared participant characteristics and exposure to Momwell’s content on different platforms in relation to whether participants completed the second survey using chi-square tests for categorical variables, Wilcoxon rank sum tests for poor physical and mental health days, and *t* tests (2-tailed) for other continuous variables.

### Planned Analyses

The aims of this project are to (1) describe how perinatal persons and mothers engage with psychoeducational content and community; (2) describe the perceived benefits of exposure to and engagement with psychoeducational content and community; (3) examine associations between engagement with digital psychoeducational content and maternal mental health, parenting attitudes, and interparental relationships; and (4) examine changes in mental health and parenting attitudes and concurrent engagement with digital psychoeducational content and community over 2 to 3 months. We have several planned analyses corresponding to these aims; we will report the results in future publications.

First, we will describe how perinatal persons and mothers engage with psychoeducational content and community. We will describe exposure to Momwell’s psychoeducational content and engagement with the Momwell community over the past 4 weeks using frequencies and percentages. Engagement metrics include duration of exposure (<6 months, ≥6 but <12 months, and at least 12 months); frequency of exposure to content on any digital platform (not at all in the past 4 weeks, 1-2 times, ≥3 times but not daily, and daily); and how often participants listened to the podcast, read the blog, read posts, watched videos on social media, liked or reacted to social media content, and replied to social media content (not at all in the past 4 weeks, 1-2 times, ≥3 times but not daily, and daily).

Second, we will describe the perceived benefits of exposure to and engagement with the content and community. We will report participants’ degree of agreement with statements about changes in their lives since joining the Momwell community using frequencies and percentages. As participants who joined the Momwell community in the past 7 days were not asked these questions, the analyses will not include these participants. We will conduct sensitivity analyses of the item about perceived impact on partner or coparent communication including only participants who reported that their children lived with 2 parents in the same household and mothers in cohort 1 who reported that they had a parenting partner. We will conduct content analyses [55] of responses to open-ended questions on the main and second survey about what Momwell social media post, blog post, or podcast episode had the biggest impact on participants’ lives.

Third, we will examine associations between engagement with digital psychoeducational content and maternal mental health, parenting attitudes, and interparental relationships. We will summarize measures of depressive symptoms, anxiety symptoms, parenting efficacy and satisfaction, and interpersonal relations using means and SDs overall and in relation to engagement metrics. We will compare these outcomes in relation to engagement metrics using crude and adjusted linear regression models. Potential confounders will include sociodemographic, household, and health-related characteristics. Analyses of parenting attitudes and interparental relationships will be limited to participants in cohort 1 as only these participants completed these measures.

Fourth, we will examine changes in mental health and parenting attitudes and concurrent engagement with digital psychoeducational content and community over 2 to 3 months. We will calculate changes in mental health and parenting outcomes from the main survey to the second survey among participants who completed both surveys. We will compare changes in these outcomes in relation to engagement metrics reported on the second survey using crude and adjusted linear regression models. Potential confounders will include sociodemographic, household, and health-related characteristics. Analyses of changes in parenting attitudes and interparental relationships will be limited to participants in cohort 1.

## Results

### Recruitment and Data Collection

**Figure 1:**
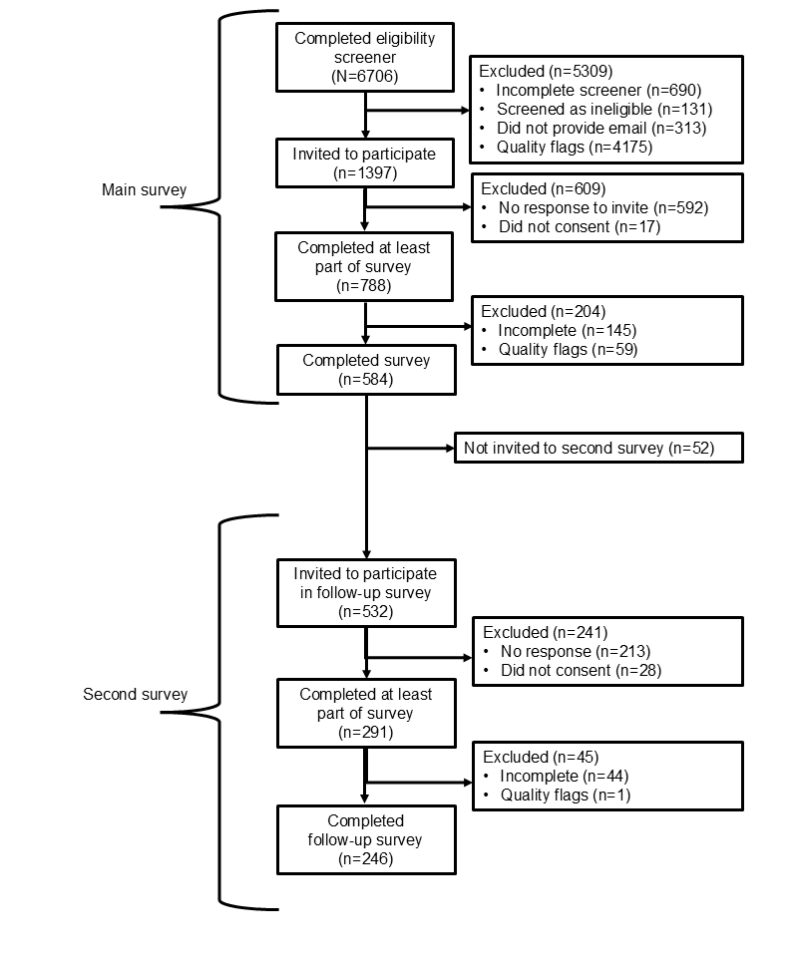
Participant flow during recruitment and data collection.

Participant flow during recruitment and data collection is shown in Figure 1. Recruitment, eligibility screening, and completion of the main survey took place from July 2023 to September 2023. Of the 6706 eligibility screeners, 690 (10.3%) were incomplete, 131 (2%) were screened as ineligible, 313 (4.7%) were eligible but did not provide their email address, and 4175 (62.3%) were excluded during quality review (n=707, 16.9% with duplicate email addresses; n=1746, 41.8% with nonsense email addresses; and n=2052, 49.1% with conflicting information regarding digital platform engagement or recruitment source); we invited 1397 (20.8%) to participate. The participation rate (ie, the number consented divided by the number invited to participate) for the main survey was 56.4% (788/1397), and the completion rate (ie, the number who completed the survey up to the last page divided by the number consented) was 81.5% (642/788). Our final analytic sample comprised 584 perinatal persons and mothers who completed the main survey (ie, at least 80% of the questions) and were not removed during quality review (n=298, 51% in cohort 1 and n=286, 49% in cohort 2; Figure 1). Of these 584 participants, 532 (91.1%) were invited to complete an optional second survey 2 to 3 months later, from October 2023 to December 2023. For the optional second survey, the participation rate was 54.7% (291/532), and the completion rate was 83.8% (244/291). Our final analytic sample for the optional second survey was 246 participants who completed it (ie, at least 80% of the questions) and were not removed during quality review (n=149, 60.6% in cohort 1 and n=97, 39.4% in cohort 2; Figure 1).

### Sample Characteristics

In total, 0.3% (1/286) of the participants in cohort 2 indicated that they would prefer not to report their gender; all other participants identified as female. The characteristics of the sample, overall and by study cohort, are shown in Table 2. All but 2 participants (582/584, 99.7%) were mothers (the remaining 2/584, 0.3% were pregnant but did not identify as mothers). Almost all participants in cohort 1 (291/296, 98.3%) reported that they had a parenting partner; 97.6% (283/290) of parenting partners were the participants’ spouses or committed partners who lived in same household. A total of 10.8% (31/286) of cohort 2 resided in countries other than the United States and Canada, including the United Kingdom (6/286, 2.1%), Australia (5/286, 1.7%), Japan (2/286, 0.7%), and 18 other countries (1/286, 0.3% each).

**Table 2:** Characteristics of the sample overall and by study cohort (N=584).

		Overall (n=584)	Cohort 1 (n=298)	Cohort 2 (n=286)
**Identified as mothers, n (%)**		582 (99.7)	296 (99.3)	286 (100)
	Children aged 0-5 y	563 (96.4)	285 (95.6)	278 (97.2)
	Children aged 6-12 y	99 (17)	57 (19.1)	42 (14.7)
	Children aged 13-17 y	22 (3.8)	10 (3.4)	12 (4.2)
**Perinatal status, n (%)**		266 (45.5)	150 (50.3)	116 (40.6)
	Currently pregnant	59 (10.1)	32 (10.7)	27 (9.4)
	Currently post partum	210 (36)	120 (40.3)	90 (31.5)
Age (y), mean (SD)		34.2 (3.9)	34.4 (3.9)	34.1 (3.8)
**Country of residence, n (%)**				
	Canada	208 (35.6)	116 (38.9)	92 (32.2)
	United States	345 (59.1)	182 (61.1)	163 (57)
	Another country	31 (5.3)	0 (0)	31 (10.8)
Identified as a visible minority, n (%)		77 (13.2)^a^	39 (13.1)	38 (13.3)^b^
**Urbanicity of residence, n (%)**				
	Large city	150 (25.8)^c^	83 (27.9)^d^	67 (23.5)^b^
	Suburb near a large city	212 (36.4)^c^	104 (35)^d^	108 (37.9)^b^
	Small city or town	157 (27)^c^	74 (24.9)^d^	83 (29.1)^b^
	Rural area	63 (10.8)^c^	36 (12.1)^d^	27 (9.5)^b^
**Educational level, n (%)**				
	Lower than bachelor’s degree	76 (13)	38 (12.8)	38 (13.3)
	Bachelor’s (4-year university degree)	255 (43.7)	122 (40.9)	133 (46.5)
	Graduate degree	253 (43.3)	138 (46.3)	115 (40.2)
**Employment status, n (%)**				
	Working full time	284 (48.6)	138 (46.3)	146 (51.1)
	Working part time	91 (15.6)	50 (16.8)	41 (14.3)
	Working but currently on leave	74 (12.7)	40 (13.4)	34 (11.9)
	Stay-at-home mother	105 (18)	54 (18.1)	51 (17.8)
	Other employment status	30 (5.1)	16 (5.4)	14 (4.9)
**Difficulty paying for basic expenses, n (%)**				
	Not at all difficult	333 (57.1)^a^	163 (54.9)^d^	170 (59.4)
	A little difficult	170 (29.2)^a^	86 (29)^d^	84 (29.4)
	Somewhat or very difficult	80 (13.7)^a^	48 (16.2)^d^	32 (11.2)
Food insecurity, n (%)		63 (10.8)	34 (11.4)	29 (10.1)
Impaired health literacy, n (%)		95 (16.3)^c^	48 (16.1)	47 (16.5)^e^
Had physical or mental health conditions that required regular medical visits, n (%)		151 (25.9)^c^	85 (28.5)	66 (23.2)^e^
Days in the previous 4 weeks when physical health was not good, median (IQR)		0 (0-3)	0 (0-3)	0 (0-3)
Days in the previous 4 weeks when mental health was not good, median (IQR)		5 (2-10)	4 (2-8)	5 (2-10)
**Social media and podcast use, n (%)**				
	Instagram	569 (97.4)	287 (96.3)	282 (98.6)
	Facebook	516 (88.4)	266 (89.3)	250 (87.4)
	TikTok	139 (24)^f^	64 (21.5)^d^	75 (26.6)^g^
	Podcasts	312 (53.7)^h^	162 (54.7)^i^	150 (52.6)^b^

^a^n=583.

^b^n=285.

^c^n=582.

^d^n=297.

^e^n=284.

^f^n=579.

^g^n=282.

^h^n=581.

^i^n=296.

### Exposure to Momwell’s Psychoeducational Content and Community

Most of the sample (552/584, 94.5%) reported that they followed Momwell on Instagram (Table 3). Approximately 4 in 10 participants listened to the Momwell podcast (258/584, 44.2%) and received the email newsletter (240/584, 41.1%). Smaller proportions followed Momwell on Facebook (89/584, 15.2%) or TikTok (36/584, 6.2%), were a part of the Momwell Facebook group (41/584, 7%), or read the blog (156/584, 26.7%; Table 3). The largest subgroup—a third (193/584, 33%)—followed Momwell on Instagram and did not connect on any other digital platform. Other common combinations of platforms included Instagram and podcast (66/584, 11.3%); Instagram, podcast, and email newsletters (57/584, 9.8%); Instagram and email newsletters (41/584, 7%); and Instagram, podcast, blog, and email newsletters (36/584, 6.2%).

**Table 3:** Exposure to the Momwell psychoeducational content and community by digital platform and survey cohort (N=584).

	Overall, n (%)	Cohort 1 (n=298), n (%)	Cohort 2 (n=286), n (%)
Instagram	552 (94.5)	275 (92.3)	277 (96.9)
Facebook	89 (15.2)	46 (15.4)	43 (15)
Facebook group	41 (7)	19 (6.4)	22 (7.7)
TikTok	36 (6.2)	18 (6)	18 (6.3)
Podcast	258 (44.2)	129 (43.3)	129 (45.1)
Blog	156 (26.7)	76 (25.5)	80 (28)
Email newsletter	240 (41.1)	127 (42.6)	113 (39.5)

Most had been exposed to the Momwell psychoeducational content and community on each digital platform for at least 6 months: 87% (480/552) on Instagram, 81% (72/89) on Facebook, 66% (27/41) on the Facebook group, 44% (16/36) on TikTok, 59.7% (154/258) via the podcast, 65.4% (102/156) on the blog, and 84.2% (202/240) via the email newsletters. Most of the sample (433/584, 74.1%) had been following Momwell on at least one digital platform for at least 12 months, and 34.9% (204/584) had started following Momwell on a new digital platform within the previous 6 months. Nearly all participants (568/584, 97.3%) followed Momwell on at least one social media platform, and 54.5% (318/584) listened to the Momwell podcast or read their blog. A total of 30% (175/584) were exposed to Momwell content on any platform daily, and 52.4% (306/584) were exposed to content ≥3 times per week but not daily. A quarter of participants (140/563, 24.9% [n=4 missing] who had joined the Momwell community >7 days before) had obtained paid services from Momwell—6% (34/564) had purchased a guided journal or other self-paced tool from Momwell, 16.3% (92/566) had enrolled in a Momwell workshop or course, and 10.4% (59/567) had enrolled in therapy with a Momwell therapist.

### Characteristics of Participants Who Completed the Optional Second Survey

Participant characteristics and exposure to Momwell content on different digital platforms were largely similar among participants who completed just the main survey compared to those who also completed the second survey (*P*>.05 in all cases). Participants who completed the second survey were more likely to have chronic health conditions (75/246, 30.5% vs 76/336, 22.6%; *P*=.03) and more likely to read the Momwell blog (82/246, 33.3% vs 74/338, 21.9%; *P*=.002) than participants who did not complete the second survey. Country of residence also differed by completion of the optional second survey (102/246, 41.5% from Canada; 136/246, 55.3% from the United States; and 8/246, 3.3% from other countries among those who completed the second survey vs 106/338, 31.4% from Canada; 209/338, 61.8% from the United States; and 23/338, 6.8% from other countries among those who did not complete the second survey; *P*=.01).

## Discussion

### Principal Findings

The overall goal of this study is to understand how mothers and the perinatal population consume and engage with digital psychoeducational content and the peer community and how it impacts their mental health and parenting. In this paper, we describe the study design, including recruitment procedures, characteristics of the sample, and participants’ exposure to maternal mental health psychoeducational content on different digital platforms. We also outline planned analyses corresponding to the overall aims of the project.

While we recruited a cohort of nearly 600 pregnant persons and mothers, we encountered challenges during recruitment, particularly related to fraudulent and low-quality responses. Bots and fraudulent respondents are becoming more and more of a problem when recruiting participants online, particularly when using public recruitment links and recruiting for studies that provide participants with a monetary incentive for completing a survey [21-24]. As some of our recruitment messages were posted publicly, we recognized the potential for attracting the attention of bots or individuals trying to participate under false pretenses—and, indeed, we experienced significant interest in our study from suspicious actors (human or bots), with several hundred respondents with duplicate email addresses; >1800 with nonsense email addresses; and >2000 with conflicting information in terms of location, digital platform engagement, or recruitment source. Bot and fraud detection features of survey administration platforms such as Qualtrics and REDCap provide a starting point, but researchers must go further to verify the eligibility and quality of respondents. As recommended [23,24], we used a multifaceted, multistage strategy. We separated eligibility screening from the survey and only sent individual survey invitations to individuals who passed our reviews. We compared information collected through multiple methods (eg, recruitment source reported by participants vs recorded by Qualtrics) and the consistency of information provided by participants (eg, ages of children). Our strategy for identifying low-quality or inattentive respondents included an attention-check question, reviewing responses to open-ended survey questions, and checking for straight-lining when completing psychological measures [23,24]. We also phrased eligibility questions to reduce the chance that respondents could guess the “right” (ie, eligible) response. We followed up with participants with inconsistent information on the survey via phone or email to clarify information. While it is possible that we excluded some eligible individuals, our quality control procedures were designed to prevent bots or fraudulent participants from taking part in our study. Researchers who are recruiting samples online using public survey links are encouraged to develop and implement similar procedures for preventing and detecting bot and fraudulent respondents. For additional discussion and specific recommendations, researchers are encouraged to read the work by Walker et al [23] and Wang et al [24], especially their table 1 and tables 2 and 4, respectively.

Another recruitment challenge was related to which members of the Momwell community volunteered to participate in this study. We had originally intended to invite only new followers or subscribers (ie, those who had started following Momwell in the previous 7 days) to complete the optional second survey to explore engagement and concurrent changes in mental health and parenting self-efficacy soon after becoming exposed to Momwell’s psychoeducational content and community. However, only 42 eligible respondents reported joining Momwell within the previous 7 days (of whom n=15, 36% completed the main survey)—perhaps a result of algorithmic biases regarding which followers were more likely to see our recruitment messages [56,57] and which followers were interested in sharing their experiences with us. Therefore, we pivoted our design to invite any participants to complete the second survey.

In total, 13.2% (77/584) of our sample identified as a visible minority compared to 31% of women aged 25 to 64 years in Canada [58]; 48% of birthing persons in the United States identify as a race or ethnicity other than non-Hispanic White [59]. In our sample, food insecurity was reported by 10.8% (63/584), which is similar to food insecurity among households with children and married couples in the United States (11%) [60] but lower than the proportion among women aged 25 to 44 years in Canada (20%) [61]. Our sample was more likely to have a bachelor’s degree or higher education (508/584, 87%) than birthing persons in the United States (36%) [59] and women aged 25 to 34 years born in Canada (40%) [62]. However, in the United States, use of Instagram and Facebook is higher among women with higher levels of education (34% of those with at most a high school education, 58% of those with some college education or an associate’s degree, and 52% of those with a bachelor’s degree or higher education for Instagram and 71%, 84%, and 82%, respectively, for Facebook) [14]. Similarly, listening to podcasts is more common among adults in the United States with higher levels of education (59% of those with at most a high school education, 64% of those with some college education or an associate’s degree, and 79% of those with a bachelor’s degree or higher education among adults aged 18-29 years and 44%, 58%, and 70%, respectively, among adults aged 30-49 years) [18]. Whether these differences are a function of the demographics of the Momwell community or due to which Momwell followers volunteered to participate in this study is unknown. Future studies could estimate this by including engagement with digital platforms generally and with specific communities (such as Momwell) in population-based studies of perinatal persons and mothers. Future studies could also use specific strategies to recruit a more diverse sample.

### Limitations

This study has additional limitations worth noting. First, our sample is composed of perinatal persons and mothers who volunteered to participate in this study and, thus, likely does not represent all individuals who consume Momwell content. Specifically, more engaged followers (eg, those who regularly comment on or like Instagram posts) are more likely to have seen our recruitment messages in their social media feeds [56,57], and those who regularly read the weekly email newsletters or listen to the podcast are more likely to have seen our recruitment messages disseminated through these communication channels. Followers or community members who feel more connected with the community or perceive higher benefit may have been more likely to volunteer to share their experiences, and thus, perceived impacts may overrepresent community members who perceive a greater positive impact on their lives and well-being. Another limitation is the proportion of participants who chose to complete the optional second survey (149/298, 50% of those who completed the longer survey and 97/286, 33.9% of those who completed the shorter survey). The lower response rate for participants who completed the shorter survey may be in part due to the incentive structure as these participants were entered into a lottery with 1 in 30 selected to receive a gift card. However, demographic characteristics and exposure to Momwell content on different digital platforms were overall quite similar among participants who completed just the main survey compared to those who also completed the second survey.

### Conclusions

In this paper, we describe the design and methods of this study and the characteristics of pregnant persons and mothers exposed to or who engage with Momwell’s psychoeducational content and community. In future papers, we will describe how perinatal persons and mothers engage with Momwell’s psychoeducational content and community; perinatal persons’ and mothers’ perceived impacts of this content and peer interactions; associations between exposure to and engagement with digital psychoeducational content and maternal mental health, parenting attitudes, and interparental relationships; and concurrent engagement over 2 to 3 months and changes in mental health and parenting attitudes. Beyond these planned analyses, we hope that these data can provide additional insights into the health and well-being of pregnant persons and mothers. We have shared a deidentified public use dataset with a subset of the data collected (variables omitted to protect participant confidentiality) [63], and researchers interested in collaborating with our research team are encouraged to email the first author.

Maternal mental health is critically important, and understanding how individuals, clinicians, and health systems can leverage digital platforms to disseminate evidence-based digital psychoeducational content and connect perinatal persons and mothers with mental health care professionals and peers has the potential to change how we care for individuals during these life phases. As most women of childbearing age and mothers in the United States and Canada use social media [12-15] and listen to podcasts [18], there is great potential for wide dissemination of relevant and impactful evidence-based psychoeducational content that can support maternal mental health. We have designed this study to provide insights into how perinatal persons and mothers leverage digital psychoeducational content and communities to support their mental health during pregnancy, the postpartum period, and the early years of motherhood.

## Abbreviations

PSOC: Parenting Sense of Competence Scale
REDCap: Research Electronic Data Capture

## Data Availability

A deidentified dataset with a subset of the data collected (variables omitted to protect participant confidentiality) is available in the Open Inter-university Consortium for Political and Social Research repository [63]. Researchers interested in collaborating with our research team are encouraged to email the first author.
